# Carotenoid-derived bioactive metabolites shape plant root architecture to adapt to the rhizospheric environments

**DOI:** 10.3389/fpls.2022.986414

**Published:** 2022-10-26

**Authors:** Danping Ke, Jinggong Guo, Kun Li, Yujie Wang, Xiaomeng Han, Weiwei Fu, Yuchen Miao, Kun-Peng Jia

**Affiliations:** ^1^ State Key Laboratory of Cotton Biology, Henan Joint International Laboratory for Crop Multi-Omics Research, School of Life Sciences, Henan University, Kaifeng, China; ^2^ Sanya Institute of Henan University, Sanya, Hainan, China; ^3^ Academy for Advanced Interdisciplinary Studies, Henan University, Kaifeng, China

**Keywords:** root development, carotenoids, carotenoid-derived bioactive molecules, root growth regulators, adaption

## Abstract

Roots are important plant organs for the uptake of water and nutrient elements. Plant root development is finely regulated by endogenous signals and environmental cues, which shapes the root system architecture to optimize the plant growth and adapt to the rhizospheric environments. Carotenoids are precursors of plant hormones strigolactones (SLs) and ABA, as well as multiple bioactive molecules. Numerous studies have demonstrated SLs and ABA as essential regulators of plant root growth and development. In addition, a lot carotenoid-derived bioactive metabolites are recently identified as plant root growth regulators, such as anchorene, β-cyclocitral, retinal and zaxinone. However, our knowledge on how these metabolites affect the root architecture to cope with various stressors and how they interact with each other during these processes is still quite limited. In the present review, we will briefly introduce the biosynthesis of carotenoid-derived root regulators and elaborate their biological functions on root development and architecture, focusing on their contribution to the rhizospheric environmental adaption of plants.

## Introduction

Roots are important plant organs for the uptake of water and nutrient elements. Most of the roots grow in soil, constituting the underground part and providing physical support to the aerial proportion of the plant (Petricka et al., 2012). In addition, roots have great significance for plants to cope with a variety of environmental stressors (Karlova et al., 2021). The dicotyledonous model plant Arabidopsis has a typical tap root system comprising of primary root, lateral roots, adventitous roots, and the most recently identified anchor roots. In Arabidopsis, a primary root is firstly developed from the radicle of the seed (Petricka et al., 2012). A pair of anchor roots will immediately initiate at the root-hypocotyl junction and conditionally emerge later (Jia et al., 2019b). Soon after, the lateral roots will develop successively in the primary root in a circadian manner (Moreno-Risueno et al., 2010). Additionally, the roots produced in the shoot and leaves are collectively referred to as adventitious roots (Bellini et al., 2014). The mature zone of all types of roots produces root hairs, which expands the surface of the root system. In comparison to the tap root systems in dicotyledons, monocotyledonous plants usually possess a fibrous root system that is composed of crown roots and lateral roots (Bellini et al., 2014). Different types of roots constitute the plant root system architecture (RSA), which optimizes the growth and adaption of plants (Leftley et al., 2021).

Root development and RSA are finely regulated by a variety of endogenous regulators. Plant hormones, especially auxin, play key roles in these processes (Leftley et al., 2021). During the embryonic root formation, polar auxin transport in quiescent center (QC) is involved in the basal root pole formation. And auxin participates in the whole developmental stage of lateral roots, including positioning, initiation, outgrowth and emergence (Perianez-Rodriguez et al., 2021). Moreover, auxin is also involved in the development of adventitious roots and anchor roots by the interaction with other regulators (Bellini et al., 2014; Jia et al., 2019b).

In addition to auxin, other phytohormones, such as jasmonic acid, SLs, and ABA, are also essential to the root development under specific environmental conditions (Marzec and Melzer, 2018; Mostofa et al., 2018; Zhang et al., 2019; Ramachandran et al., 2020; Brookbank et al., 2021). Jasmonic acid is a key signal to initiate the adventitious root formation in wounded non-root organs to induce plant regeneration (Zhou et al., 2019; Ikeuchi et al., 2020); SLs shape the plant RSA to make better use of nutrient elements especially under phosphorus (P)- and nitrogen (N)-starvation conditions (Marzec and Melzer, 2018; Mostofa et al., 2018); while ABA mainly modulates root growth directions toward favorable surviving environment to avoid the disadvantageous abiotic stresses in rhizosphere (Harris, 2015; Emenecker and Strader, 2020; Brookbank et al., 2021). Both SLs and ABA are derived from carotenoids, which are a class of isopronoid photosynthesis pigments and act as precursors of a serial of bioactive molecules (Schwartz et al., 1997; Alder et al., 2012). Most recently, several endogenous carotenoid-derived metabolites are identified as new regulators of root growth and development in plants, such as β-cyclocitral (Dickinson et al., 2019), anchorene (Jia et al., 2019b), iso-anchorene (Jia et al., 2021a), zaxinone (Wang et al., 2019) and retinal (Dickinson et al., 2021). Therefore, it seems that carotenoids and carotenoid-derived metabolites play intriguingly essential roles in root development and shaping RSA.

## Carotenogenesis and carotenoid metabolism

### Carotenogenesis in plants

Carotenoids are isoprenoid photosynthetic accessory pigments with significant functions in photosynthesis in plants (Maoka, 2020). They are essential for scavenging free radicals and for photoprotection to protect the photosystems from singlet oxygen damage caused by excess exposure to light (Hashimoto et al., 2016). In addition, carotenoids act as precursors of metabolites with significant biological functions, such as phytohormones SLs and ABA. Carotenoids consist of eight isoprene units with a 40-carbon skeleton (Rodriguez-Concepcion et al., 2018). In plants, they are synthesized and stored in plastids. The synthesis of carotenoids starts from the condensation of two geranylgeranyl pyrophosphat (GGPP) molecules catalyzed by phytoene synthase (PSY) to yield 15-*cis*-phytoene, which is an important rate-limiting step in carotenoids biosynthesis (Rosas-Saavedra and Stange, 2016). 15-*cis*-phytoene is further catalyzed by two desaturases and two isomerases to produce lycopene. The cyclization of lycopene is diverged into α-carotene and β-carotene branch depending on the catalysis of different cyclases. Hydroxylation of the two ionone rings in α-carotene and β-carotene by various hydroxylases leads to lutein and zeaxanthin, respectively (Fiore et al., 2006; Kim and Dellapenna, 2006). Lutein and zeaxanthin can be further isomerized in the double bonds of the chain and modified in the rings, resulting in the enormous diversity of this class of compounds (Von Lintig and Sies, 2013).

### Apocarotenoid and carotenoid-derived bioactive metabolites

**Figure 1:**
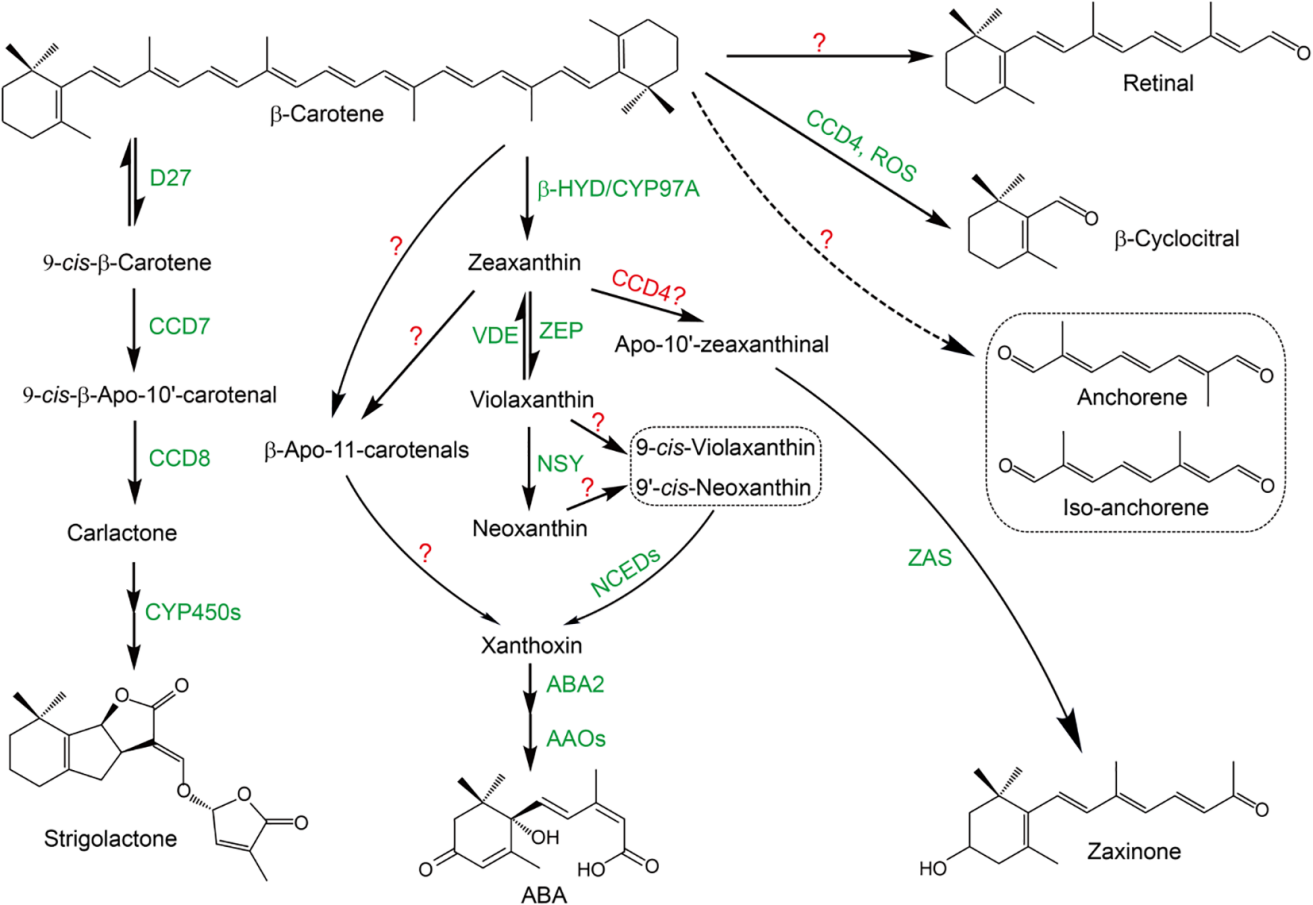
The biosynthesis pathway of carotenoid-derived root regulators. The identified enzymes are marked as green and the presumptive enzyme is marked as red. “?” indicates that the enzymes responsible for the reaction have not been identified yet. Strigolactone: β-Carotene is first isomerized into 9-*cis*-β-carotene by dwarf27 (D27) in a reversible manner; 9-*cis*-β-carotene is sequentially oxidative cleaved by CCD7 and CCD8 to produce the key intermediate carlactone, which is further catalyzed by CYP450s (OsMAX1s) to produce strigolactones. ABA: β-Carotene is hydroxylased by non-heme diiron oxidase (HYD)/CYP97A into zeaxanthin; zeaxanthin and violaxanthin can be interconverted into each other by zeaxanthin epoxidase (ZEP) and violaxanthin de-epoxidase (VDE), respectively; violaxanthin is transformed into neoxanthin by neoxanthin synthase (NSY); violaxanthin and neoxanthin are further isomerized by yet unknown enzymes into 9-*cis*-violaxanthin and 9’-*cis*-neoxanthin, respectively, which are further oxidatively cleaved by NCEDs into xanthoxin; xanthoxin is then sequentially catalyzed by aba-deficient 2 (ABA2) and abscisic aldehyde oxidases (AAOs) to finally produce ABA; recently, an alternative route from β-carotene or zeaxanthin to xanthoxin has been identified, which uses β-apo-11-carotenals as intermediates, even though the exact enzymes responsible for this route have not been identified. Zaxinone: ZAS catalyzes the presumptive zeaxanthin oxidative cleavage product, apo-10’-zeaxanthinal to produce zaxinone. β-Cyclocitral can be produced from β-carotene catalyzed by CCD4 or non-enzymatically by ROS. Principally, retinal, anchorene and iso-anchorene could be produced from the oxidative cleavage of β-carotene, however, the experimental evidence is still lacking.

Owing to the extended conjugated double bond system in their structure, carotenoids are prone to be cleaved by carotenoid cleavage dioxygenase (CCD) and/or non-enzymatically by reactive oxygen species (ROS), leading to a diverse family of important metabolites, apocarotenoids (Walter and Strack, 2011; Beltran and Stange, 2016; Hou et al., 2016). Apocarotenoids act as important bioactive molecules in plants, such as phytohormones ABA and SLs, and various plant growth regulators β-cyclocitral, anchorene, zaxinone, and so on (**Figure 1**) (Felemban et al., 2019; Moreno et al., 2021; Wang et al., 2021b).

Six major CCD sub-families, main contributors in carotenoids metabolism, have been identified in plants: CCD1, CCD4, CCD7, CCD8, 9-cisepoxycarotenoid dioxygenase (NCED) and zaxinone synthase (ZAS) (Wang et al., 2019; Zhang et al., 2021). CCD1 enzymes possess wide substrate and double-bond specificity to produce a large number of volatiles and dialdehyde products, indicating a role for them in scavenging destructed carotenoids (Ilg et al., 2009). CCD4 enzymes specifically cleave the C9-C10 or C7-C8 double bonds in bicyclic carotenoids, which have essential biological functions in regulating carotenoid contents and pigmentation in different plant organs and tissues (Zheng et al., 2021). In addition, it is believed that CCD4 is responsible for the production of β-cyclocitral (Ilg et al., 2009; Bruno et al., 2016), even though ROS have also been shown to be involved in this process, likely by a non-enzymatically cleavage manner (**Figure 1**) (Ramel et al., 2012). The biological functions of CCD7 and CCD8 subfamilies have been widely investigated, which are key enzymes responsible for the biosynthesis of SLs (**Figure 1**) (Alder et al., 2012; Jia et al., 2018). NCEDs are rate-limiting enzymes in ABA biosynthesis pathway, catalyzing the stereospecific cleavage of 9-cis-epoxycarotenoids to produce the key ABA intermediate xanthoxin (**Figure 1**) (Schwartz et al., 1997; Nambara and Marion-Poll, 2005). ZAS sub-family members were most recently reported to catalyze the production of zaxinone, a newly identified plant growth regulator in rice (**Figure 1**) (Wang et al., 2019). Need to notice, ZAS orthologues are only present in genomes of arbuscular mycorrhizal fungi (AMF) host species, suggesting an important role of ZAS subfamily in arbuscular mycorrhizal symbiosis (Wang et al., 2019).

Among these carotenoid-derived bioactive metabolites, SLs and ABA display significant physiological activities in regulating root growth and development (Mostofa et al., 2018; Brookbank et al., 2021). In addition, several newly identified bioactive apocarotenoids were demonstrated as important plant root growth regulators. Retinal, which was previously characterized as key precursor of vitamin A in mammals, is identified as conserved endogenous signal in Arabidopsis to regulate periodic oscillatory lateral root initiation (**Figure 1**) (Dickinson et al., 2021); anchorene is specifically involved in anchor root formation in Arabidopsis (**Figure 1**) (Jia et al., 2019b); β-cyclocitral and iso-anchorene affect root apical meristem to regulate primary root growth in Arabidopsis (**Figure 1**) (Dickinson et al., 2019; Jia et al., 2021a); while zaxinone was shown to affects root biomass and nutrient utilization efficiency in rice (Wang et al., 2019). Obviously, carotenoid-derived bioactive metabolites play essential roles underground to regulate root growth and development. In the following part, we will elaborate how these carotenoid-derived bioactive metabolites regulate root growth in plants to adapt to the adverse rhizospheric environments.

## Biological functions of carotenoid-derived root growth regulators

### SLs shape root architecture to adapt to P and N nutrient deficiency

**Figure 2:**
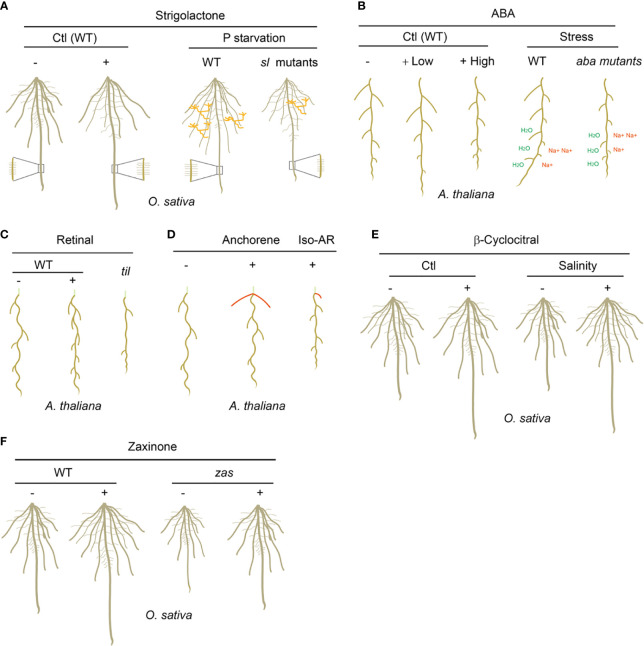
The biological functions of carotenoid-derived metabolites on plant root development and adaption to rhizospheric environments. **(A)** SLs enhance the length of the primary root, seminal roots and root hairs, but inhibit lateral root density in rice under normal growth condition; under P starvation growth condition, wild type (WT) rice plants grow longer seminal roots and root hairs, and less lateral roots, which is not observed in SL biosynthesis and signaling mutants (*sl* mutants); WT rice plants establish more arbuscular mycorrhizal symbiosis than *sl* mutants under P starvation. AMF are marked as orange. **(B)** Low concentrations of ABA promote the length of primary root and lateral roots, while high concentrations of ABA inhibit them; under drought and salinity soil growth conditions, the WT but not the ABA biosynthesis and signaling mutants (*aba* mutants) plants possess hydrotropism and negative halotropism manners to grow toward higher water potentials and escape from salty environment. “H_2_O” indicates higher water potentials and “Na^+^” indicates higher salt concentrations. **(C)** Retinal determines the spatial distribution of lateral root prebranch sites in a periodic oscillation transcriptional mechanism in Arabidopsis; *til* mutant has significantly decreased-lateral root capacity and -root clock amplitude due to the decreased sensitivity to retinal. **(D)** Anchorene promotes the formation of anchor roots, while iso-anchorene (iso-AR) mainly inhibits the growth of primary root. Anchor roots are marked as red and the partial hypocotyl is marked as light green. **(E)** β-Cyclocitral enhances primary root growth and lateral root branching by promoting root stem cell divisions; under salinity growth condition, exogenous applied β-cyclocitral could promote the root growth and therefore increase the tolerance to salt stress. **(F)** Zaxinone promotes the root growth and enhances the root biomass in rice; the *zas* mutant displayed decreased root biomass which can be restored by zaxinone application. “-” indicates no treatment and “+” indicates exogenous carotenoid-derived metabolites treatments. “Ctl” indicates plants grow under normal condition.

SLs are identified as novel carotenoid-derived plant hormones that shape plant shoot architecture in 2008 (Gomez-Roldan et al., 2008; Umehara et al., 2008). Previously, SLs were mainly characterized as plant secreting rhizospheric signals to promote AMF hyphal branching and stimulate the germination of parasite weeds (Al-Babili and Bouwmeester, 2015; Jia et al., 2018). Afterwards, SLs were identified as key regulators in root architecture in different species (**Figure 2A**). In rice and Arabidopsis, SL biosynthesis and signaling deficient mutants developed more lateral roots and shorter primary roots compared to wild type plants under optimal growth conditions, suggesting SLs promote the primary root growth but inhibit the lateral root formation (Koltai, 2011; Ruyter-Spira et al., 2011; Ruyter-Spira et al., 2013). Consistently, exogenously applied SL analog GR24 restored the lateral- and primary-root phenotypes in SL biosynthesis mutants but not in SL signaling mutants in both rice and Arabidopsis (Koltai, 2011; Ruyter-Spira et al., 2011). Further studies showed that the enhanced root growth upon SL application correlates with increased meristem cortical cell number and transition zone size in primary root. SLs also mediate adventitious root development even the effects vary in different species. SLs suppress the formation of adventitious root in Arabidopsis and pea (Rasmussen et al., 2012), while promote crown root growth and adventitious root elongation in rice (Sun et al., 2015). In addition, SLs have a significantly promoting effect on the root hair elongation (Kapulnik et al., 2011).

P and N are two of the macro nutrient elements required for plant growth and development. Under P- or N- starvation growth conditions, the plants are prone to grow a shallow RSA with longer lateral roots and shorter primary roots, which is believed to facilitate nutrient absorption efficiency (Marzec and Melzer, 2018; Hu et al., 2021; Liu, 2021; Pélissier et al., 2021). Although the molecular mechanism of how P- and N-starvation shape root architecture is complex, SLs seem play essential roles in the adaptive responses to P- and N-deficiency of plants. In rice, P- or N-starvation lead to increased seminal root length and decreased lateral root density in wild type seedlings, while these effects are largely repressed in SL biosynthesis and signaling mutants (**Figure 2A**) (Koltai, 2011; Ruyter-Spira et al., 2011; Mayzlish-Gati et al., 2012; Sun et al., 2014). Further studies demonstrate that P- or N-starvation rapidly induce the expression of SL biosynthesis genes, such as CCD7 and CCD8, and increase the biosynthesis of SLs (Yoneyama et al., 2012; Sun et al., 2014). The increased SL contents contribute to the adaption of the plants to P- or N-starvation at least in two aspects. 1) SLs work together with other signals to shape the RSA (longer seminal roots and root hairs) for better nutrient utilization efficiency; 2) Elevated SL biosynthesis in roots will increase the SL content in root exudates, which will promote the symbiotic relationship between host plants and AMF by inducing AMF hyphae branching, and therefore further enhance the adaptive responses of plants toward nutrient deficiency. The effect of SLs on nutrient starvation responses has great potential for developing strategies to improve crop nutrient utilization efficiency and productivity in unfertile soils.

Auxin is a master regulator on the root development in plants (Petricka et al., 2012). The root growth is determined by the auxin concentration gradient along the longitudinal axis of root meristem, which is mainly maintained by the action of auxin efflux transporter PIN proteins (Blilou et al., 2005; Paponov et al., 2005). It has been demonstrated that SL signaling acts upstream of auxin in regulating primary root growth, as well as lateral root initiation and emergence (Kapulnik et al., 2011; Koltai, 2011; Mayzlish-Gati et al., 2012; Sun et al., 2014). The regulation of lateral root density by SLs could attribute to the change of lateral auxin reflux in roots (Cheng et al., 2013). In addition, SL application on Arabidopsis roots declines PIN1, PIN2, PIN3 and PIN7 proteins levels in primary root tips, which enhances the auxin accumulation in primary root tips and ultimately results in increased primary root growth (Ruyter-Spira et al., 2011; Pandya-Kumar et al., 2014).

### ABA directs root growth to cope with various abiotic stresses

ABA is another carotenoid derived plant hormone (Nambara and Marion-Poll, 2005; Jia et al., 2021b). ABA regulates multiple growth and developmental processes including seed dormancy and germination, stomatal movement, plant senescence, as well as responses to various abiotic stresses (Brookbank et al., 2021). In addition, ABA plays a key role on plant root growth and development (**Figure 2B**) (Harris, 2015). During seed dormancy, the presence of endogenous ABA causes the arrest of root apical meristem (RAM) which will resume functioning upon the removal of ABA (Lopez-Molina et al., 2001). During seedling development, ABA promotes the maintenance of stem cells in the RAM by maintaining the quiescence of QC; at the same time, ABA could increase the differentiated cell size in the RAM (Zhang et al., 2010; Takatsuka and Umeda, 2019). Therefore, low concentrations of ABA promote the primary root growth, and reduced primary root length was observed in the ABA biosynthesis- and signaling-mutants due to a smaller differentiated cell size (Rosales et al., 2019); while application of high concentrations of exogenous ABA inhibits primary root growth by inhibiting the stem cell division in the RAM (**Figure 2B**) (Rosales et al., 2019). ABA has a similar effect but different sensitivity on lateral roots in comparison to the primary root (De Smet et al., 2003). ABA also stimulates the production of root hairs by reducing the length of root hairs but increasing their density (Salazar-Henao et al., 2016; Rymen et al., 2017). Thus, appropriate ABA content is necessary for the normal development of plant root system.

Drought and salinity are two widespread abiotic stressors for plants, which provoked water deficit and nutrient imbalance. ABA signaling mediates the root architecture and morphology to cope with these stressors. Drought stress could promote the primary root growth but inhibit the lateral root growth by enhancing the accumulation of ABA in roots (Pierik and Testerink, 2014; Rosales et al., 2019). Therefore, under drought stress plants accumulate ABA in the roots which results in fewer axial/lateral roots and a deeper root to allow for efficient water capture and thereby ameliorates drought stress. It was believed that the accumulation of ABA in roots is mainly transported from aboveground part under drought stress condition (McAdam et al., 2016; Xie et al., 2020). While challenged with salt stress, the growth of primary roots and lateral roots will be arrested, which likely requires the fast ABA biosynthesis in the roots (Chen et al., 2012). The ABA accumulation in the roots under salt stress will inhibit the cell differentiation in the QC, and therefore protect the RAM. Furthermore, it was found that lateral roots are more sensitive to salinity compared with primary roots (Duan et al., 2013; Geng et al., 2013). On the other hand, ABA mediates root growth directions to cope with various stress signals. Hydrotropism and negative halotropism are representatives of root responses to drought and salt stresses, respectively, which enable guided root growth in soil (**Figure 2B**). Hydrotropism is the bending of the root to grow toward higher water potentials, which depends on the core ABA signal transduction pathway in cortical cells of the root elongation zone in Arabidopsis (Pierik and Testerink, 2014; Miao et al., 2021). In contrast, ABA synthesis or ABA signaling defective mutants have a significantly reduced hydrotropic response (Karlova et al., 2021; Miao et al., 2021). Negative halotropism means root bending away from salty environment, which is a key strategy for plant root to avoid high salinity stress. As mentioned above, high salinity stress can arrest the growth of primary- and lateral-roots due to the accumulation of ABA (Pierik and Testerink, 2014; Karlova et al., 2021). Therefore, negative halotropism will guide the root grow away from areas of high salinity environment.

It has been shown that ABA mediated root growth is related to various hormone signals and ROS (Verma et al., 2016; Emenecker and Strader, 2020; Brookbank et al., 2021). ROS functions as a secondary messenger for root cell division and elongation. ROS accumulation inhibits the elongation of primary roots. ABA stimulates the root elongation rate by reducing ROS accumulation (Yang et al., 2014). In addition, ABA have been shown to interact with auxin signaling in controlling hydropatterning which directs lateral roots grow toward higher water availability (Bao et al., 2014; Robbins and Dinneny, 2018; Emenecker and Strader, 2020). The accumulation of ABA under low water potential decreases the local auxin biosynthesis, therefore inhibits lateral root formation (Emenecker and Strader, 2020). This mechanism may also explain how meristems are maintained and protected under stress conditions or during dormancy.

### Retinal is an endogenous carotenoid-derived metabolite involved in the lateral root clock

The formation of lateral roots plays a decisive role in the root architecture. Lateral roots initiate from the determined xylem pole pericycle cells which were called lateral root founder cells along the root axis (Banda et al., 2019). Lateral root founder cells undergo a stereotypic division patterning to form lateral root primordia which will emerge to form lateral roots later conditionally (Banda et al., 2019). The emergence of lateral root primordia is influenced by various environmental cues, such as nutrient status, temperature, water availability (Muller et al., 2019); whereas, the establishment of lateral root pre-patterning along the root axis has been reported to be determined by a periodic oscillation transcriptional mechanism in Arabidopsis, which is known as the root clock (Moreno-Risueno et al., 2010). The root clock establishes lateral root prebranch sites through oscillating gene expression approximately every 6 hours. The root clock and primary root growth jointly determine the spatial distribution of lateral root prebranch sites (Moreno-Risueno et al., 2010; Van Norman et al., 2013).

It was believed that the developmental pre-patterning of lateral roots requires a carotenoid-derived metabolite signal, because repression of the carotenoid biosynthesis through either chemical inhibitors or genetic manipulation results in the depletion of the root clock (Van Norman et al., 2014). Further evidence indicates the putative β-carotene derived signal is rather than ABA or SLs. Therefore, an uncharacterized apocarotenoid likely serves as a regulator for lateral root prebranch sites and coordinates the lateral root clock with the development of primordia (Van Norman et al., 2014). Using biochemistry and genetic techniques, Dickinson et al., ambiguously demonstrated that retinal, which could be produced from the cleavage of β-carotene, acts as a root clock signal in Arabidopsis (**Figure 2C**) (Dickinson et al., 2021). Dickinson et al., further identified temperature induced lipocalin (TIL) as a specific retinal binding protein in Arabidopsis. Interestingly, TIL shows significant homology with a vertebrate retinal binding lipocalin, retinol binding protein 4 (RBP4). Consistently, retinal also works on the occupational somitogenesis clock in vertebrate, which suggests a potential conserved biological role of retinal in plants and vertebrate. *TIL* expression well overlaps the root clock signal in spatial and temporal patterns (Dickinson et al., 2021). Moreover, *Arabidopsis til* mutant has significantly decreased-lateral root capacity, -root clock amplitude and -sensitivity to retinal (**Figure 2C**). Moreover, TIL specifically binds to retinal in a heterologous system. In addition, TIL was previously identified as a protein involved in heat stress tolerance, which suggests the root clock might be mediated by temperature. Indeed, heat shock stress significantly decreases the amplitude of the root clock, and the temperature sensitivity of the root clock is suppressed in the *til* mutant, indicating TIL is important for proper response to heat stress during lateral root organogenesis (Dickinson et al., 2021).

Taken together, retinal is a new carotenoid-derived bioactive metabolite that specifically mediates oscillatory lateral root initiation through a potential TIL regulated signaling pathway in Arabidopsis, which is essential for heat stress mediated lateral root organogenesis.

### Anchorene specifically promotes anchor root formation in Arabidopsis

Anchor roots were recently identified as a new kind of roots that grow just in the root hypocotyl junction site in Arabidopsis (**Figure 2D**) (Jia et al., 2019b; Pérez-Pérez, 2020). Different from lateral roots and adventitious roots, anchor roots initiate from the xylem pole pericycle cells as early as 3 days post germination of Arabidopsis seeds. In Arabidopsis Col-0 ecotype, the seedlings have very little frequency (< 5%) to grow anchor roots under optimal growth condition. However, when grown in poor nutrient sandy soil or primary root tips are excised, the seedlings have much higher frequency (>50%) to grow anchor roots, suggesting anchor roots are important for primary root replacement and nutrient acquisition (Jia et al., 2019b).

Jia et al., further demonstrated that a carotenoid-derived metabolite is required for the anchor root formation, because repression of the carotenoid biosynthesis through either chemical inhibitors or genetic manipulation suppresses anchor root formation. By screening a serial of apocarotenoids theoretically derived from carotenoids, Jia et al., identified anchorene as a specific regulator of anchor root formation. Exogenous anchorene application fully restored the anchor root formation in carotenoid biosynthesis deficient plants (**Figure 2D**). Moreover, anchorene can be detected in plants, and its amount was significantly reduced in carotenoid biosynthesis repression plants, indicating anchorene is an endogenous carotenoid-derived metabolite (Jia et al., 2019b; Mi et al., 2019). In addition, under N starvation growth condition, the Arabidopsis seedlings grew more anchor roots and accumulated more anchorene, suggesting anchorene is important for the plants to adapt to N starvation (Jia et al., 2019b). Need to notice, iso-anchorene is another carotenoid-derived metabolite which is a structural isomer of anchorene. However, iso-anchorene specifically inhibits the primary root growth, which provides an interesting example that chemicals with similar structures have quite different physiological functions (**Figure 2D**) (Jia et al., 2021a).

Auxin plays an essential role in anchor root development, since anchor root formation is strongly induced by the auxin analog NAA, but impeded by the auxin transport inhibitor NPA (Jia et al., 2019b). Further studies demonstrate that anchorene regulates anchor root development mainly through the modulation of auxin homeostasis. Anchorene application significantly enhances the auxin signaling in both anchor root primordia and their surrounding tissues. Consistently, anchorene treatment resulted in a significant increase of PIN3 and LAX3 proteins, which are the auxin efflux and influx transporters, respectively. In addition, anchorene application increased the transcript levels of several auxin biosynthetic genes, including two key genes involved in tryptophan-dependent auxin biosynthesis, *TAA1* and *YUC7*. Therefore, Jia et al., demonstrates anchorene as an important carotenoid-derived bioactive metabolite that regulates anchor root formation and N starvation adaption in Arabidopsis. Interestingly, anchorene can be detected in other species, such as rice, tomato and pepper; moreover, anchorene can promote crown root growth in rice, suggesting anchorene is a conserved root growth regulator in higher plants (Jia et al., 2019b; Mi et al., 2019). However, the precise physiological functions of anchorene in other species are still need to be investigated.

### β-Cyclocitral enhances root growth and branching to adapt to various abiotic stresses

β-Cyclocitral was previously characterized as an oxidized by-product of β-carotene cleaved by CCD1 or CCD4, mediating an important protective retrograde response that reduces the levels of toxic ROS under oxidative stress (Ramel et al., 2012; Havaux, 2020). Later, β-Cyclocitral was identified as an efficient root growth regulators in different plant species, which was found to enhance primary root growth and lateral root branching by promoting root stem cell divisions (**Figure 2E**) (Dickinson et al., 2019). While the Arabidopsis *ccd1ccd4* biosynthesis mutants displayed reduced root stem cell divisions, which could be restored by the application of β-Cyclocitral, indicating CCD1 and CCD4 are likely involved in β-Cyclocitral mediated root growth (Dickinson et al., 2019). In rice and tomato, β-cyclocitral displayed a conserved effect on the promotion of root growth (**Figure 2E**). Moreover, β-cyclocitral treatment enhanced plant vigor in rice when exposed to salt-contaminated soil, which has important implication potentials in agriculture (**Figure 2E**) (Dickinson et al., 2019). In addition, the potential β-cyclocitral derivative β-cyclocitral acid was shown to be involved in drought stress responses in Arabidopsis (D'Alessandro et al., 2019; Havaux, 2020). This proves β-cyclocitral as a broadly effective root growth promoter in both monocots and dicots and a valuable tool to enhance crop vigor under environmental stress.

The molecular mechanism of how β-cyclocitral regulates root development is still not clear. However, it seems that β-cyclocitral mediated root growth is independent of auxin, brassinosteroid, and ROS signaling pathways (Dickinson et al., 2019).

### Zaxinone promotes root growth and controls arbuscular mycorrhizal colonization in rice

ZAS, a member of the least characterized CCD subfamily, can specifically cleave the apo-10’-zeaxanthinal to yield a novel apocarotenoid metabolite zaxinone *in vitro* (Wang et al., 2019). A rice loss-of-function *zas* mutant contained less zaxinone in root tissues, displaying reduction in the main crown root length and number of crown roots. The root phenotype of *zas* mutant can be restored by the application of zaxinone, indicating a specific role of zaxinone and ZAS on the root growth of rice (**Figure 2F**) (Wang et al., 2019). Further examination at the cellular levels of the root supplied by zaxinone demonstrates that zaxinone increases in apex length, diameter, and the number of cells and cortex cell layers in rice (Wang et al., 2021a). ZAS subfamily members are not present in mustard family species according to the phylogenetic analyses; therefore Arabidopsis does not have a ZAS homolog. Interestingly, zaxinone can be detected in Arabidopsis plants and exogenous application of zaxinone on Arabidopsis seedlings inhibits the primary root growth, suggesting a different effect of zaxinone on root development in Arabidopsis compared with rice (Ablazov et al., 2020). However, due to the lack of ZAS homologs in Arabidopsis, these different effects might result from the dosage effect of zaxinone.

Further studies show that *zas* mutant has elevated expression of SL biosynthesis genes and increased levels of SLs (Wang et al., 2019). Application of zaxinone can decrease SL content in roots and root exudates. Moreover, the root growth promoting effect of exogenously applied zaxinone was not observed in SL deficient mutants, suggesting the bioactivity of zaxinone likely requires functional SL biosynthesis and signaling. SLs are essential for the germination of parasitic weeds, such as *Striga* and *Phelibanche*, severe threats to global food security (Ruyter-Spira et al., 2013; Jia et al., 2019a); therefore, the repression of SLs in root exudates by zaxinone could relieve the infection of *Striga*. In addition, zaxinone seems play an important role in arbuscular mycorrhizal symbiosis, since ZAS orthologues are absent in genomes of non-AMF host species (Fiorilli et al., 2019). Recently, reduced arbuscular mycorrhizal colonization was observed in the *zas* mutant in rice, which could not be rescued by the application of zaxinone (Votta et al., 2022). Further evidence demonstrated that ZAS acts in a regulatory network that involves SLs activity, which leads to a different effect on arbuscular mycorrhizal symbiosis from that of exogenous zaxinone application (Votta et al., 2022). Taken together, zaxinone and ZAS play important roles in root development and biotic interactions, which has great potentials for increasing crop growth and combating parasitic weeds.

## Conclusions and perspectives

Carotenoids are important source of known and postulated hormones and signaling molecules in plants. Carotenogenesis is also essential for normal plant root growth and development. Indeed, a lot studies have unraveled the molecular mechanism of how carotenoid-derived plant hormones SLs and ABA regulate root development and shape RSA to adapt to the rhizospheric environments. However, more carotenoid-derived root regulators have recently been identified and investigated, such as anchorene, retinal, zaxinone and β-cyclocitral. And our knowledge on how these metabolites are produced and affect the root architecture is still quite limited. In addition, even though all these metabolites originate from the same metabolic pathway, the relationships between all these molecules and their interaction with other hormones known to be involved in the root development are largely elusive. In the future, the characterization of genes responsible for the production and modification of these metabolites, and for the signaling transduction of them will largely expand the understanding of the molecular mechanism of these metabolites in regulating root development, which will also push forward the potential application of them in agriculture.

On the other side, several carotenoid-derived metabolites, such as SLs, ABA and zaxinone, have also been reported or suggested to be released into rhizosphere to play essential communication signals with other organism. The study on the biological functions of root released carotenoid-derived metabolites will open another gate for the significance of these metabolites in the future.

## Author contributions

K-PJ and DK conceived the work and wrote the manuscript. All the authors read and revised the manuscript. All authors contributed to the article and approved the submitted version.

## Funding

This work was supported by the National Natural Science Foundation of China (32170271), the Project of Sanya Yazhou Bay Science and Technology City (SCKJ-JYRC-2022-19), Natural Science Foundation of Henan Province (222300420024).

## Acknowledgments

We apologize to colleagues whose work was not referred due to space limitations.

## Conflict of interest

The authors declare that the research was conducted in the absence of any commercial or financial relationships that could be construed as a potential conflict of interest.

## Publisher’s note

All claims expressed in this article are solely those of the authors and do not necessarily represent those of their affiliated organizations, or those of the publisher, the editors and the reviewers. Any product that may be evaluated in this article, or claim that may be made by its manufacturer, is not guaranteed or endorsed by the publisher.

## Abbreviations

SLs, Strigolactones; RSA, Root system architecture; QC, Quiescent center; N, Nitrogen; P, Phosphorus; ROS, Reactive oxygen species; AMF, Arbuscular mycorrhizal fungi; RAM, Root apical meristem; WT, Wild type; ABA, Abscisic acid; GGPP, Geranylgeranyl pyrophosphate; NAA, 1-naphthlcetic acid; NPA, 1-N-naphthylphthalamic acid; Iso-AR, Iso-anchorene; PSY, Phytoene synthase; CCD, Carotenoid cleavage dioxygenase; ZAS, Zaxinone synthase; TIL, Temperature induced lipocalin; RBP4, Retinol binding protein 4; D27, Dwarf27; CYP450, Cytochrome p450; OsMAX1, Oryza sativa more axillary 1; HYD, Non-heme diiron oxidase; CYP97A, Cytochrome p450-type monooxygenase 97a; ZEP, Zeaxanthin epoxidase; VDE, Violaxanthin de-epoxidase; NSY, Neoxanthin synthase; ABA2, Aba-deficient 2; AAOs, Abscisic aldehyde oxidases; NCED, 9-cisepoxycarotenoid dioxygenase; PIN, Pin-formed family protein; LAX3, Like aux1 3; TAA1, Tryptophan aminotransferase of arabidopsisArabidopsis 1; YUC7, Yucca 7.

## References

[B1] Ablazov A. Mi J. Jamil M. Jia K. P. Wang J. Y. Feng Q.. (2020). The apocarotenoid zaxinone is a positive regulator of strigolactone and abscisic acid biosynthesis in arabidopsis roots. Front. Plant Sci. 11, 578. doi: 10.3389/fpls.2020.00578 32477389 PMC7240130

[B2] Al-Babili S. Bouwmeester H. J. (2015). Strigolactones, a novel carotenoid-derived plant hormone. Annu. Rev. Plant Biol. 66, 161–186. doi: 10.1146/annurev-arplant-043014-114759 25621512

[B3] Alder A. Jamil M. Marzorati M. Bruno M. Vermathen M. Bigler P.. (2012). The path from β-carotene to carlactone, a strigolactone-like plant hormone. Science 335, 1348–1351. doi: 10.1126/science.1218094 22422982

[B4] Banda J. Bellande K. Von Wangenheim D. Goh T. Guyomarc'h S. Laplaze L.. (2019). Lateral root formation in arabidopsis: A well-ordered LRexit. Trends Plant Sci. 24, 826–839. doi: 10.1016/j.tplants.2019.06.015 31362861

[B5] Bao Y. Aggarwal P. Robbins N. E. Sturrock 2. C. J. Thompson M. C. Tan H. Q.. (2014). Plant roots use a patterning mechanism to position lateral root branches toward available water. Proc. Natl. Acad. Sci. U.S.A. 111, 9319–9324. doi: 10.1073/pnas.1400966111 24927545 PMC4078807

[B6] Bellini C. Pacurar D. I. Perrone I. (2014). Adventitious roots and lateral roots: similarities and differences. Annu. Rev. Plant Biol. 65, 639–666. doi: 10.1146/annurev-arplant-050213-035645 24555710

[B7] Beltran J. C. Stange C. (2016). Apocarotenoids: A new carotenoid-derived pathway. Subcell Biochem. 79, 239–272. doi: 10.1007/978-3-319-39126-7_9 27485225

[B8] Blilou I. Xu J. Wildwater M. Willemsen V. Paponov I. Friml J.. (2005). The PIN auxin efflux facilitator network controls growth and patterning in arabidopsis roots. Nature 433, 39–44. doi: 10.1038/nature03184 15635403

[B9] Brookbank B. P. Patel J. Gazzarrini S. Nambara E. (2021). Role of basal ABA in plant growth and development. Genes 12, 1936. doi: 10.3390/genes12121936 34946886 PMC8700873

[B10] Bruno M. Koschmieder J. Wuest F. Schaub P. Fehling-Kaschek M. Timmer J.. (2016). Enzymatic study on AtCCD4 and AtCCD7 and their potential to form acyclic regulatory metabolites. J. Exp. Bot. 67, 5993–6005. doi: 10.1093/jxb/erw356 27811075 PMC5100015

[B11] Cheng X. Ruyter-Spira C. Bouwmeester H. (2013). The interaction between strigolactones and other plant hormones in the regulation of plant development. Front. Plant Sci. 4. doi: 10.3389/fpls.2013.00199 PMC3683633 23785379

[B12] Chen J. H. Jiang H. W. Hsieh E. J. Chen H. Y. Chien C. T. Hsieh H. L.. (2012). Drought and salt stress tolerance of an arabidopsis glutathione s-transferase U17 knockout mutant are attributed to the combined effect of glutathione and abscisic acid. Plant Physiol. 158, 340–351. doi: 10.1104/pp.111.181875 22095046 PMC3252094

[B13] D'Alessandro S. Mizokami Y. Légeret B. Havaux M. (2019). The apocarotenoid β-cyclocitric acid elicits drought tolerance in plants. iScience 19, 461–473. doi: 10.1016/j.isci.2019.08.003 31437750 PMC6710299

[B14] De Smet I. Signora L. Beeckman T. Inzé D. Foyer C. H. Zhang H. (2003). An abscisic acid-sensitive checkpoint in lateral root development of arabidopsis. Plant J. 33, 543–555. doi: 10.1046/j.1365-313X.2003.01652.x 12581312

[B15] Dickinson A. J. Lehner K. Mi J. Jia K. P. Mijar M. Dinneny J.. (2019). Beta-cyclocitral is a conserved root growth regulator. Proc. Natl. Acad. Sci. U.S.A. 116, 10563–10567. doi: 10.1073/pnas.1821445116 31068462 PMC6534974

[B16] Dickinson A. J. Zhang J. Luciano M. Wachsman G. Sandoval E. Schnermann M.. (2021). A plant lipocalin promotes retinal-mediated oscillatory lateral root initiation. Science 373, 1532–1536. doi: 10.1126/science.abf7461 34446443 PMC8827267

[B17] Duan L. Dietrich D. Ng C. H. Chan P. M. Bhalerao R. Bennett M. J.. (2013). Endodermal ABA signaling promotes lateral root quiescence during salt stress in arabidopsis seedlings. Plant Cell 25, 324–341. doi: 10.1105/tpc.112.107227 23341337 PMC3584545

[B18] Emenecker R. J. Strader L. C. (2020). Auxin-abscisic acid interactions in plant growth and development. Biomolecules 10, 281. doi: 10.3390/biom10020281 PMC7072425 32059519

[B19] Felemban A. Braguy J. Zurbriggen M. D. Al-Babili S. (2019). Apocarotenoids involved in plant development and stress response. Front. Plant Sci. 10, 1168. doi: 10.3389/fpls.2019.01168 31611895 PMC6777418

[B20] Fiore A. Dall'osto L. Fraser P. D. Bassi R. Giuliano G. (2006). Elucidation of the beta-carotene hydroxylation pathway in arabidopsis thaliana. FEBS Lett. 580, 4718–4722. doi: 10.1016/j.febslet.2006.07.055 16890225

[B21] Fiorilli V. Wang J. Y. Bonfante P. Lanfranco L. Al-Babili S. (2019). Apocarotenoids: Old and new mediators of the arbuscular mycorrhizal symbiosis. Front. Plant Sci. 10. doi: 10.3389/fpls.2019.01186 PMC6776609 31611899

[B22] Geng Y. Wu R. Wee C. W. Xie F. Wei X. Chan P. M.. (2013). A spatio-temporal understanding of growth regulation during the salt stress response in arabidopsis. Plant Cell 25, 2132–2154. doi: 10.1105/tpc.113.112896 23898029 PMC3723617

[B23] Gomez-Roldan V. Fermas S. Brewer P. B. Puech-Pagès V. Dun E. A. Pillot J. P.. (2008). Strigolactone inhibition of shoot branching. Nature 455, 189–194. doi: 10.1038/nature07271 18690209

[B24] Harris J. M. (2015). Abscisic acid: Hidden architect of root system structure. Plants (Basel) 4, 548–572. doi: 10.3390/plants4030548 27135341 PMC4844405

[B25] Hashimoto H. Uragami C. Cogdell R. J. (2016). Carotenoids and photosynthesis. Subcell Biochem. 79, 111–139. doi: 10.1007/978-3-319-39126-7_4 27485220

[B26] Havaux M. (2020). β-cyclocitral and derivatives: Emerging molecular signals serving multiple biological functions. Plant Physiol. Biochem. 155, 35–41. doi: 10.1016/j.plaphy.2020.07.032 32738580

[B27] Hou X. Rivers J. Leon P. Mcquinn R. P. Pogson B. J. (2016). Synthesis and function of apocarotenoid signals in plants. Trends Plant Sci. 21, 792–803. doi: 10.1016/j.tplants.2016.06.001 27344539

[B28] Hu Q. Q. Shu J. Q. Li W. M. Wang G. Z. (2021). Role of auxin and nitrate signaling in the development of root system architecture. Front. Plant Sci. 12. doi: 10.3389/fpls.2021.690363 PMC8631788 34858444

[B29] Ikeuchi M. Rymen B. Sugimoto K. (2020). How do plants transduce wound signals to induce tissue repair and organ regeneration? Curr. Opin. Plant Biol. 57, 72–77. doi: 10.1016/j.pbi.2020.06.007 32738736

[B30] Ilg A. Beyer P. Al-Babili S. (2009). Characterization of the rice carotenoid cleavage dioxygenase 1 reveals a novel route for geranial biosynthesis. FEBS J. 276, 736–747. doi: 10.1111/j.1742-4658.2008.06820.x 19120446

[B31] Jia K. P. Baz L. Al-Babili S. (2018). From carotenoids to strigolactones. J. Exp. Bot. 69, 2189–2204. doi: 10.1093/jxb/erx476 29253188

[B32] Jia K. P. Dickinson A. J. Mi J. Cui G. Xiao T. T. Kharbatia N. M.. (2019b). Anchorene is a carotenoid-derived regulatory metabolite required for anchor root formation in arabidopsis. Sci. Adv. 5, eaaw6787. doi: 10.1126/sciadv.aaw6787 31807696 PMC6881154

[B33] Jia K. P. Li C. Bouwmeester H. J. Al-Babili S. (2019a). “Strigolactone biosynthesis and signal transduction,” in Strigolactones - biology and applications. Eds. Koltai H. Prandi. C. (Cham: Springer International Publishing), 1–45. doi: 10.1007/978-3-030-12153-2_1

[B34] Jia K. P. Mi J. Ablazov A. Ali S. Yang Y. Balakrishna A.. (2021a). Iso-anchorene is an endogenous metabolite that inhibits primary root growth in arabidopsis. Plant J. 107, 54–66. doi: 10.1111/tpj.15271 33837613

[B35] Jia K. P. Mi J. Ali S. Ohyanagi H. Moreno J. C. Ablazov A.. (2021b). An alternative, zeaxanthin epoxidase-independent abscisic acid biosynthetic pathway in plants. Mol. Plant. 15, 151–166. doi: 10.1016/j.molp.2021.09.008 34547513

[B36] Kapulnik Y. Resnick N. Mayzlish-Gati E. Kaplan Y. Wininger S. Hershenhorn J.. (2011). Strigolactones interact with ethylene and auxin in regulating root-hair elongation in arabidopsis. J. Exp. Bot. 62, 2915–2924. 21307387 10.1093/jxb/erq464

[B37] Karlova R. Boer D. Hayes S. Testerink C. (2021). Root plasticity under abiotic stress. Plant Physiol. 187, 1057–1070. doi: 10.1093/plphys/kiab392 34734279 PMC8566202

[B38] Kim J. Dellapenna D. (2006). Defining the primary route for lutein synthesis in plants: the role of arabidopsis carotenoid beta-ring hydroxylase CYP97A3. Proc. Natl. Acad. Sci. U.S.A. 103, 3474–3479. doi: 10.1073/pnas.0511207103 16492736 PMC1413914

[B39] Koltai H. (2011). Strigolactones are regulators of root development. New Phytol. 190, 545–549. doi: 10.1111/j.1469-8137.2011.03678.x 21638793

[B40] Leftley N. Banda J. Pandey B. Bennett M. Voß U. (2021). Uncovering how auxin optimizes root systems architecture in response to environmental stresses. Cold Spring Harb. Perspect. Biol. 13, a040014. doi: 10.1101/cshperspect.a040014 33903159 PMC8559545

[B41] Liu D. (2021). Root developmental responses to phosphorus nutrition. J. Integr. Plant Biol. 63, 1065–1090. doi: 10.1111/jipb.13090 33710755

[B42] Lopez-Molina L. Mongrand S. Chua N. H. (2001). A postgermination developmental arrest checkpoint is mediated by abscisic acid and requires the ABI5 transcription factor in arabidopsis. Proc. Natl. Acad. Sci. U.S.A. 98, 4782–4787. doi: 10.1073/pnas.081594298 11287670 PMC31911

[B43] Maoka T. (2020). Carotenoids as natural functional pigments. J. Nat. Med. 74, 1–16. doi: 10.1007/s11418-019-01364-x 31588965 PMC6949322

[B44] Marzec M. Melzer M. (2018). Regulation of root development and architecture by strigolactones under optimal and nutrient deficiency conditions. Int. J. Mol. Sci. 19, 1887. doi: 10.3390/ijms19071887 PMC6073886 29954078

[B45] Mayzlish-Gati E. De-Cuyper C. Goormachtig S. Beeckman T. Vuylsteke M. Brewer P. B.. (2012). Strigolactones are involved in root response to low phosphate conditions in arabidopsis. Plant Physiol. 160, 1329–1341. doi: 10.1104/pp.112.202358 22968830 PMC3490576

[B46] McAdam S. A. Brodribb T. J. Ross J. J. (2016). Shoot-derived abscisic acid promotes root growth. Plant Cell Environ. 39, 652–659. doi: 10.1111/pce.12669 26514625

[B47] Miao R. Yuan W. Wang Y. Garcia-Maquilon I. Dang X. Li Y.. (2021). Low ABA concentration promotes root growth and hydrotropism through relief of ABA INSENSITIVE 1-mediated inhibition of plasma membrane h(+)-ATPase 2. Sci. Adv. 7, 1–14. doi: 10.1126/sciadv.abd4113 PMC7968848 33731345

[B48] Mi J. Jia K. P. Balakrishna A. Feng Q. Al-Babili S. (2019). A highly sensitive SPE derivatization-UHPLC-MS approach for quantitative profiling of carotenoid-derived dialdehydes from vegetables. J. Agric. Food Chem. 67, 5899–5907. doi: 10.1021/acs.jafc.9b01749 31055928 PMC7722347

[B49] Moreno J. C. Mi J. Alagoz Y. Al-Babili S. (2021). Plant apocarotenoids: from retrograde signaling to interspecific communication. Plant J. 105, 351–375. doi: 10.1111/tpj.15102 33258195 PMC7898548

[B50] Moreno-Risueno M. A. Van Norman J. M. Moreno A. Zhang J. Ahnert S. E. Benfey P. N. (2010). Oscillating gene expression determines competence for periodic arabidopsis root branching. Science 329, 1306–1311. doi: 10.1126/science.1191937 20829477 PMC2976612

[B51] Mostofa M. G. Li W. Nguyen K. H. Fujita M. Tran L. P. (2018). Strigolactones in plant adaptation to abiotic stresses: An emerging avenue of plant research. Plant Cell Environ. 41, 2227–2243. doi: 10.1111/pce.13364 29869792

[B52] Muller B. Guédon Y. Passot S. Lobet G. Nacry P. Pagès L.. (2019). Lateral roots: Random diversity in adversity. Trends Plant Sci. 24, 810–825. doi: 10.1016/j.tplants.2019.05.011 31320193

[B53] Nambara E. Marion-Poll A. (2005). Abscisic acid biosynthesis and catabolism. Annu. Rev. Plant Biol. 56, 165–185. doi: 10.1146/annurev.arplant.56.032604.144046 15862093

[B54] Pandya-Kumar N. Shema R. Kumar M. Mayzlish-Gati E. Levy D. Zemach H.. (2014). Strigolactone analog GR24 triggers changes in PIN2 polarity, vesicle trafficking and actin filament architecture. New Phytol. 202, 1184–1196. doi: 10.1111/nph.12744 24571327

[B55] Paponov I. A. Teale W. D. Trebar M. Blilou I. Palme K. (2005). The PIN auxin efflux facilitators: evolutionary and functional perspectives. Trends Plant Sci. 10, 170–177. doi: 10.1016/j.tplants.2005.02.009 15817418

[B56] Pélissier P. M. Motte H. Beeckman T. (2021). Lateral root formation and nutrients: nitrogen in the spotlight. Plant Physiol. 187, 1104–1116. doi: 10.1093/plphys/kiab145 33768243 PMC8566224

[B57] Pérez-Pérez J. M. (2020). Anchor root development: A world within worlds. Mol. Plant 13, 1105–1107. doi: 10.1016/j.molp.2020.07.005 32682964

[B58] Perianez-Rodriguez J. Rodriguez M. Marconi M. Bustillo-Avendaño E. Wachsman G. Sanchez-Corrionero A.. (2021). An auxin-regulable oscillatory circuit drives the root clock in arabidopsis. Sci. Adv. 7, eabd4722. doi: 10.1126/sciadv.abd4722 33523850 PMC7775764

[B59] Petricka J. J. Winter C. M. Benfey P. N. (2012). Control of arabidopsis root development. Annu. Rev. Plant Biol. 63, 563–590. doi: 10.1146/annurev-arplant-042811-105501 22404466 PMC3646660

[B60] Pierik R. Testerink C. (2014). The art of being flexible: how to escape from shade, salt, and drought. Plant Physiol. 166, 5–22. doi: 10.1104/pp.114.239160 24972713 PMC4149730

[B61] Ramachandran P. Augstein F. Nguyen V. Carlsbecker A. (2020). Coping with water limitation: Hormones that modify plant root xylem development. Front. Plant Sci. 11. doi: 10.3389/fpls.2020.00570 PMC7243681 32499804

[B62] Ramel F. Birtic S. Ginies C. Soubigou-Taconnat L. Triantaphylidès C. Havaux M. (2012). Carotenoid oxidation products are stress signals that mediate gene responses to singlet oxygen in plants. Proc. Natl. Acad. Sci. U.S.A. 109, 5535–5540. doi: 10.1073/pnas.1115982109 22431637 PMC3325660

[B63] Rasmussen A. Mason M. G. De Cuyper C. Brewer P. B. Herold S. Agusti J.. (2012). Strigolactones suppress adventitious rooting in arabidopsis and pea. Plant Physiol. 158, 1976–1987. doi: 10.1104/pp.111.187104 22323776 PMC3320200

[B64] Robbins N. E. 2nd Dinneny J. R. (2018). Growth is required for perception of water availability to pattern root branches in plants. Proc. Natl. Acad. Sci. U.S.A. 115, E822–E831. doi: 10.1073/pnas.1710709115 29317538 PMC5789911

[B65] Rodriguez-Concepcion M. Avalos J. Bonet M. L. Boronat A. Gomez-Gomez L. Hornero-Mendez D.. (2018). A global perspective on carotenoids: Metabolism, biotechnology, and benefits for nutrition and health. Prog. Lipid Res. 70, 62–93. doi: 10.1016/j.plipres.2018.04.004 29679619

[B66] Rosales M. A. Maurel C. Nacry P. (2019). Abscisic acid coordinates dose-dependent developmental and hydraulic responses of roots to water deficit. Plant Physiol. 180, 2198–2211. doi: 10.1104/pp.18.01546 31164395 PMC6670111

[B67] Rosas-Saavedra C. Stange C. (2016). Biosynthesis of carotenoids in plants: Enzymes and color. Subcell Biochem. 79, 35–69. doi: 10.1007/978-3-319-39126-7_2 27485218

[B68] Ruyter-Spira C. Al-Babili S. van der Krol S. Bouwmeester H. (2013). The biology of strigolactones. Trends Plant Sci. 18, 72–83. doi: 10.1016/j.tplants.2012.10.003 23182342

[B69] Ruyter-Spira C. Kohlen W. Charnikhova T. Van Zeijl A. Van Bezouwen L. De Ruijter N.. (2011). Physiological effects of the synthetic strigolactone analog GR24 on root system architecture in arabidopsis: another belowground role for strigolactones? Plant Physiol. 155, 721–734. doi: 10.1104/pp.110.166645 21119044 PMC3032462

[B70] Rymen B. Kawamura A. Schäfer S. Breuer C. Iwase A. Shibata M.. (2017). ABA suppresses root hair growth *ia* the OBP4 transcriptional regulator. Plant Physiol. 173, 1750–1762. doi: 10.1104/pp.16.01945 28167701 PMC5338652

[B71] Salazar-Henao J. E. Vélez-Bermúdez I. C. Schmidt W. (2016). The regulation and plasticity of root hair patterning and morphogenesis. Development 143, 1848–1858. doi: 10.1242/dev.132845 27246711

[B72] Schwartz S. H. Tan B. C. Gage D. A. Zeevaart J. A. Mccarty D. R. (1997). Specific oxidative cleavage of carotenoids by VP14 of maize. Science 276, 1872–1874. doi: 10.1126/science.276.5320.1872 9188535

[B73] Sun H. Tao J. Hou M. Huang S. Chen S. Liang Z.. (2015). A strigolactone signal is required for adventitious root formation in rice. Ann. Bot. 115, 1155–1162. doi: 10.1093/aob/mcv052 25888593 PMC4648462

[B74] Sun H. Tao J. Liu S. Huang S. Chen S. Xie X.. (2014). Strigolactones are involved in phosphate- and nitrate-deficiency-induced root development and auxin transport in rice. J. Exp. Bot. 65, 6735–6746. doi: 10.1093/jxb/eru029 24596173 PMC4246174

[B75] Takatsuka H. Umeda M. (2019). ABA inhibits root cell elongation through repressing the cytokinin signaling. Plant Signal Behav. 14, e1578632. doi: 10.1080/15592324.2019.1578632 30741075 PMC6422398

[B76] Umehara M. Hanada A. Yoshida S. Akiyama K. Arite T. Takeda-Kamiya N.. (2008). Inhibition of shoot branching by new terpenoid plant hormones. Nature 455, 195–200. doi: 10.1038/nature07272 18690207

[B77] Van Norman J. M. Xuan W. Beeckman T. Benfey P. N. (2013). To branch or not to branch: the role of pre-patterning in lateral root formation. Development 140, 4301–4310. doi: 10.1242/dev.090548 24130327 PMC4007709

[B78] Van Norman J. M. Zhang J. Cazzonelli C. I. Pogson B. J. Harrison P. J. Bugg T. D.. (2014). Periodic root branching in arabidopsis requires synthesis of an uncharacterized carotenoid derivative. Proc. Natl. Acad. Sci. U.S.A. 111, 17. doi: 10.1073/pnas.1403016111 PMC3977299 24639533

[B79] Verma V. Ravindran P. Kumar P. P. (2016). Plant hormone-mediated regulation of stress responses. BMC Plant Biol. 16, 86–86. doi: 10.1186/s12870-016-0771-y 27079791 PMC4831116

[B80] Von Lintig J. Sies H. (2013). Carotenoids. Arch. Biochem. Biophys. 539, 99–101. doi: 10.1016/j.abb.2013.09.014 24076362

[B81] Votta C. Fiorilli V. Haider I. Wang J. Y. Balestrini R. Petřík I.. (2022). Zaxinone synthase controls arbuscular mycorrhizal colonization level in rice. Plant J. 111, 1688–1700. doi: 10.1111/tpj.15917 35877598 PMC9543690

[B82] Walter M. H. Strack D. (2011). Carotenoids and their cleavage products: biosynthesis and functions. Nat. Prod. Rep. 28, 663–692. doi: 10.1039/C0NP00036A 21321752

[B83] Wang J. Y. Alseekh S. Xiao T. Ablazov A. Perez De Souza L. Fiorilli V.. (2021a). Multi-omics approaches explain the growth-promoting effect of the apocarotenoid growth regulator zaxinone in rice. Commun. Biol. 4, 021–02740. doi: 10.1038/s42003-021-02740-8 PMC8545949 34697384

[B84] Wang J. Y. Haider I. Jamil M. Fiorilli V. Saito Y. Mi J.. (2019). The apocarotenoid metabolite zaxinone regulates growth and strigolactone biosynthesis in rice. Nat. Commun. 10, 810. doi: 10.1038/s41467-019-08461-1 30778050 PMC6379432

[B85] Wang J. Y. Lin P. Y. Al-Babili S. (2021b). On the biosynthesis and evolution of apocarotenoid plant growth regulators. Semin. Cell Dev. Biol. 109, 3–11. doi: 10.1016/j.semcdb.2020.07.007 32732130

[B86] Xie Q. Essemine J. Pang X. Chen H. Cai W. (2020). Exogenous application of abscisic acid to shoots promotes primary root cell division and elongation. Plant Sci. 292, 23. doi: 10.1016/j.plantsci.2019.110385 32005390

[B87] Yang L. Zhang J. He J. Qin Y. Hua D. Duan Y.. (2014). ABA-mediated ROS in mitochondria regulate root meristem activity by controlling PLETHORA expression in arabidopsis. PloS Genet. 10, e1004791. doi: 10.1371/journal.pgen.1004791 25522358 PMC4270459

[B88] Yoneyama K. Xie X. Kim H. I. Kisugi T. Nomura T. Sekimoto H.. (2012). How do nitrogen and phosphorus deficiencies affect strigolactone production and exudation? Planta 235, 1197–1207. doi: 10.1007/s00425-011-1568-8 22183123 PMC3362704

[B89] Zhang S. Guo Y. Zhang Y. Guo J. Li K. Fu W.. (2021). Genome-wide identification, characterization and expression profiles of the CCD gene family in gossypium species. 3 Biotech. 11, 249. doi: 10.1007/s13205-021-02805-9 PMC8088422 33968592

[B90] Zhang H. Han W. De Smet I. Talboys P. Loya R. Hassan A.. (2010). ABA promotes quiescence of the quiescent centre and suppresses stem cell differentiation in the arabidopsis primary root meristem. Plant J. 64, 764–774. doi: 10.1111/j.1365-313X.2010.04367.x 21105924

[B91] Zhang G. Zhao F. Chen L. Pan Y. Sun L. Bao N.. (2019). Jasmonate-mediated wound signalling promotes plant regeneration. Nat. Plants 5 (5), 491–497. doi: 10.1038/s41477-019-0408-x 31011153

[B92] Zheng X. Yang Y. Al-Babili S. (2021). Exploring the diversity and regulation of apocarotenoid metabolic pathways in plants. Front. Plant Sci. 12, 787049. doi: 10.3389/fpls.2021.787049 34956282 PMC8702529

[B93] Zhou W. Lozano-Torres J. L. Blilou I. Zhang X. Zhai Q. Smant G.. (2019). A jasmonate signaling network activates root stem cells and promotes regeneration. Cell 177, 942–956. doi: 10.1016/j.cell.2019.03.006 30955889

